# Reduction of testosterone levels in *Schistosoma haematobium-* or *Schistosoma mansoni*-infected men: a cross-sectional study in two schistosomiasis-endemic areas of the Adamawa region of Cameroon

**DOI:** 10.1186/s12879-022-07195-8

**Published:** 2022-03-07

**Authors:** Hermine Boukeng Jatsa, Ulrich Membe Femoe, Calvine Noumedem Dongmo, Romuald Issiaka Ngassam Kamwa, Betrand Nono Fesuh, Louis-Albert Tchuem Tchuente, Pierre Kamtchouing

**Affiliations:** 1 Laboratory of Animal Physiology, Department of Animal Biology and Physiology, Faculty of Science, University of Yaoundé I, P.O. Box 812, Yaoundé, Cameroon; 2 Centre for Schistosomiasis and Parasitology, P.O. Box 7244, Yaoundé, Cameroon; 3 Laboratory of Parasitology and Ecology, Department of Animal Biology and Physiology, Faculty of Science, University of Yaoundé I, P.O. Box 812, Yaoundé, Cameroon; 4 Department of Biological Science, Faculty of Science, University of Maroua, P.O. Box 46, Maroua, Cameroon; 5 Laboratory of Mathematical Engineering and Information System, Department of Mathematics, National Advances School of Engineering of Yaoundé, University of Yaoundé I, P.O. Box 8390, Yaoundé, Cameroon

**Keywords:** Male genital schistosomiasis, Testosterone, Testes, *Schistosoma haematobium*, *Schistosoma mansoni*, Cameroon

## Abstract

**Background:**

The incidence of schistosomiasis‐induced male reproductive dysfunction and infertility is probably underestimated compared to female genital schistosomiasis. This study aimed to investigate the impact of *Schistosoma haematobium* or *S. mansoni* infection on the reproductive function of men of reproductive age in Tibati and Wouldé, two endemic schistosomiasis areas in the Adamawa region of Cameroon.

**Methods:**

A total of 89 men of reproductive age (range 14–56 years) from two localities were enrolled in the study, with 51 in Tibati and 38 in Wouldé. Each participant was submitted to a questionnaire to document data on sociodemographic and stream contact behaviors. A medical examination was performed to measure the testes’ circumference and evaluate genital tract pathologies. Stool and urine samples were collected and screened for the presence of *S. haematobium* or *S. mansoni* ova. Blood serum was used to assess the levels of transaminases and testosterone.

**Results:**

*Schistosoma haematobium* was present only in Tibati, with a prevalence of 31.37%. The *S. mansoni* prevalence was 3.92% at Tibati and 44.71% at Wouldé. The intensity of infection was 22.12 ± 9.57 eggs/10 mL for *S. haematobium* and 128.10 ± 3.76 epg for *S. mansoni*. Serum transaminase activity and the mean testicular circumference of Schistosoma-positive individuals were close to *Schistosoma*-negative individuals. However, the testes size was more prominent in *S. mansoni*-positive individuals than in *S. haematobium*-positive individuals (*P* < 0.05). The serum testosterone levels of *S. haematobium-* and *S. mansoni*-positive men were significantly reduced by 56.07% (*P* < 0.001) and 51.94% (*P* < 0.01), respectively, in comparison to those of *Schistosoma*-negative men. A significant and negative correlation was established between schistosomiasis and the low serum testosterone level. Male genital tract pathologies such as scrotal abnormalities, varicocele, nodular epididymis, inguinal hernia, and hydrocele were recorded in both *Schistosoma*-positive and *Schistosoma*-negative men. However, no significant link was established between schistosomiasis infection and these pathologies.

**Conclusion:**

These results demonstrated that infection with *S. haematobium* or *S. mansoni* is associated with low production of the reproductive hormone testosterone and may be a significant cause of male infertility.

## Background

Schistosomiasis is an acute and chronic disease caused by *Schistosoma* blood flukes. In Cameroon, three species of human schistosomes occur *S. haematobium, S. mansoni,* and *S. guineensis*. People are infected while exposed to infested water during routine agricultural, domestic, occupational, and recreational activities. In 2016, the global burden of schistosomiasis was estimated at 2.50 million disability-adjusted life years (DALYs). Schistosomiasis remains a major public health problem, with at least 229 million people worldwide, and almost 90% of them lived in sub-Saharan Africa, requiring preventive chemotherapy in 2018 [1]. In Cameroon, it was estimated that 47% of the population (9,484,894 million) was at risk of schistosomiasis infection and needed preventive treatment in 2010 [2]. The country’s three northern regions, the Far North, North, and Adamawa, are the most affected. Some health districts in the South-West, Littoral, West, and Centre regions showed a high prevalence of infection. Epidemiological data collected in some schools of the Adamawa region in 2006 revealed that 14.2% of the children were infected with at least one schistosome species [3]. The strategy of choice to control schistosomiasis remains preventive chemotherapy with praziquantel. School-aged children are the primary target of these therapeutic interventions since they are the most vulnerable and most affected by the infection [4]. In Cameroon, fishermen, farmers, and irrigation workers are also high-risk groups for schistosome infection, but they are not enrolled in deworming campaigns with praziquantel [3, 5]. *Schistosoma*-positive adults generally suffer from chronic schistosomiasis with severe consequences such as liver fibrosis, portal hypertension, ascites, calcification of the bladder, kidney failure, and genital dysfunction [2]. Genital schistosomiasis occurs when *Schistosoma* circulating eggs invade the reproductive tissues of the host. It has been associated with female and male reproductive health problems such as ectopic pregnancies, abortion, low sperm count, coitus pain, vaginal bleeding, genital organ enlargement and infertility [6–8]. Female genital schistosomiasis (FGS) is well documented, and its prevalence is considerable in *S. haematobium* endemic areas (33 to 75%) [6, 9–12]. In contrast to FGS, male genital schistosomiasis (MGS) is underreported, and its burden is underestimated. *S. haematobium* and *S. mansoni* are the two species involved in MGS. The main findings reported in patients who have traveled or lived in schistosomiasis endemic areas are swelling of the scrotum and other genital organs, dilatation, and calcification of the prostate and seminal vesicles, haemospermia, hydrocele, changes in semen/ejaculate, infertility, and urethral discharge [7, 13]. Case reports have mentioned the presence of *S. haematobium* or *S. mansoni* eggs in male genital organs and semen of some young and adult patients [14–24]. Variation in the levels of sex hormones was also reported in some male patients with *S. haematobium* and/or *S. mansoni* [25–29].

In Cameroon, investigations on the association of schistosomiasis and the male reproductive tract have drawn no attention from researchers. To the best of our knowledge, this is the first report on male genital schistosomiasis in Cameroon. This study aimed to investigate the impact of infection with *S. haematobium* or *S. mansoni* on the reproductive tract of men of reproductive age in Tibati and Wouldé, two endemic schistosomiasis areas in the Adamawa region of Cameroon.

## Methods

### Study area

**Fig. 1:**
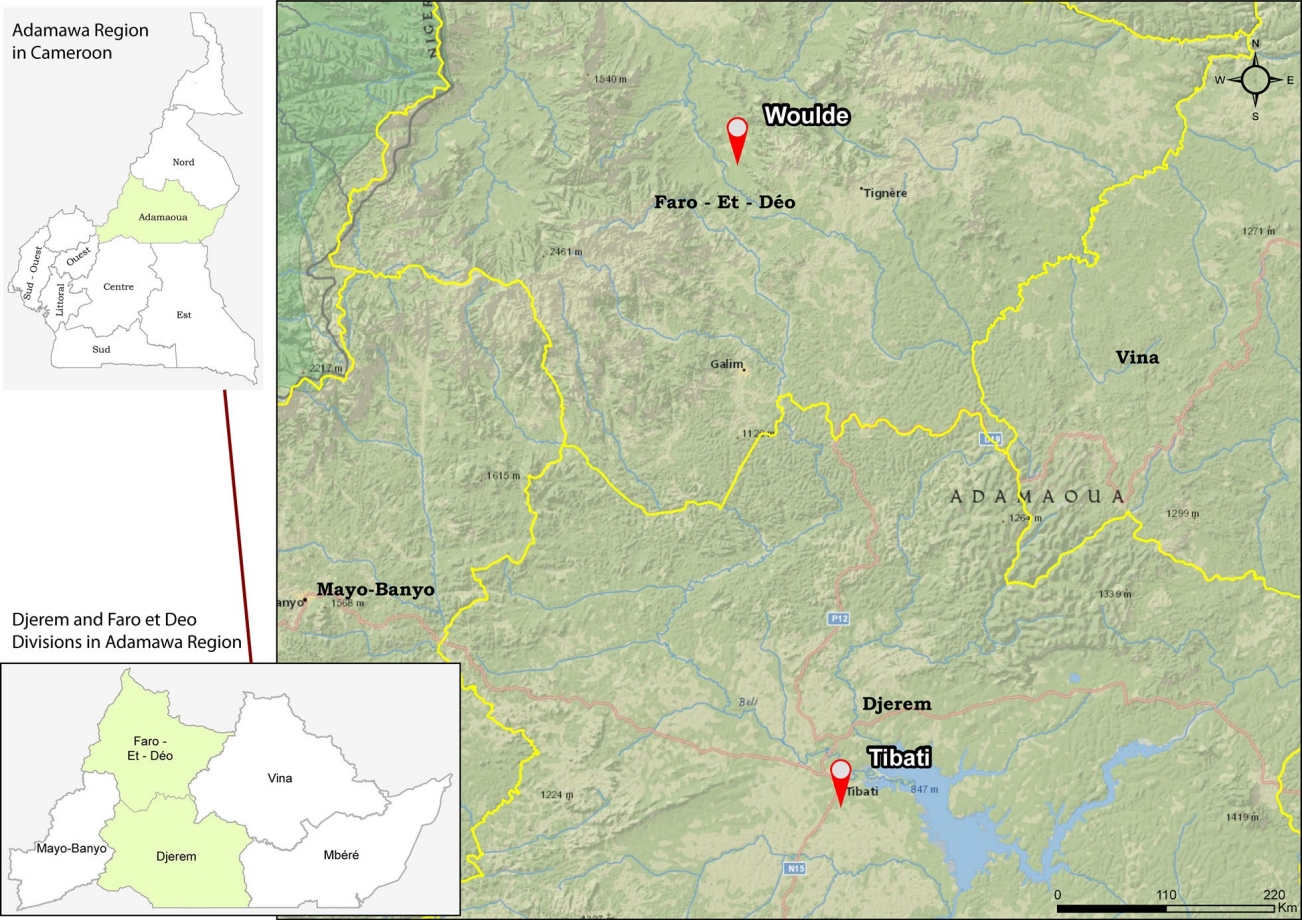
Study area: Tibati and Wouldé

Cameroon is divided into ten regions at the first level, 58 divisions at the second level, and 360 subdistricts at the third level. According to the United Nations, the population of Cameroon was estimated to be 25,876,380 inhabitants in 2019, with 1,200,970 inhabitants in the Adamawa region [30]. The Adamawa region is divided into five divisions: Vina, Mbéré, Mayo Banyo, Djerem and Faro et Déo. Tibati, the capital of the “Djerem” division, is situated between 6° 47′ N and 12° 63′ E. It is a semi-urban area with a population density of 7 inhabitants/km^2^. Located in the “Pagneré” neighborhood, Lake Pagneré is the largest in the city. Trading, fishing, and fish mongering are the main activities in Tibati. Wouldé is a village in the Mayo-Baléo subdistrict in the “Faro et Déo” division. It is situated between 7° 43′ N and 12° 47′ E and is surrounded by volcanic hills. At the entrance of this rural area flows the Wouldé River with its thermal source. The population density is 6.6 inhabitants/km^2^, and the major occupations in this community are farming, livestock, and trading. The vegetation of the Adamawa region falls within the rainforest-savannah mosaic, and the climate is a sudano-guinean type with an annual rainfall of 1500 mm. Schistosomiasis transmission presumably occurs in lakes, rivers, ponds, and marshy fields. Fulbe and Kutin ethnic groups are predominant in Tibati and Wouldé, and they are mainly Muslims. Therefore, we localized Tibati and Wouldé on the study area map conceived and realized by our research team (Fig. 1). Tibati and Wouldé (Adamawa region) belong to the *S. haematobium* and *S. mansoni* transmission zones. In addition, *S. guineensis* is found in a few localities of the Far North, North, Centre, South, Littoral, and South West [3].

### Ethical considerations

This study was approved by the National Ethics Committee of Cameroon (No. 072/CNE/DNM/08). Surveys were conducted in the communities with the approval of administrative authorities. The study’s objectives were explained to the populations, and each participant signed informed consent. When a participant was under 16 years old, written informed consent was obtained from a parent or guardian. The results were sent to each participant at the end of the study. Each *Schistosoma*-positive person was treated with praziquantel 600 mg/mL at the dose of 40 mg/kg per day orally in two divided doses for one day.

### Study design

The people involved in the study were all male volunteers of reproductive age who were residents of Lake Pagneré in Tibati and of the “Chefferie” neighborhood in Wouldé. In this study, we included non-adult men under 18 years old (14 to 17 years) when they were sexually active and were in couple with their girlfriends. Thoughtfully ill individuals showing incapacitation were not included in the study. A questionnaire was administered to each participant for personal information, fertility status, and history of sexually transmissible infections. In addition, participants underwent physical examination by a male medical practitioner to assess palpation genital tract pathologies such as varicocele, hydrocele, nodular epididymis, scrotal abnormalities, and inguinal hernia. The circumference of the two testes was also measured. Initially planned in the study, sperm collection to search for the presence of schistosome ova and conduct semen analysis was not carried out because of cultural considerations (masturbation being a taboo in those localities). Instead, urine and stool samples and blood were collected from each participant for parasitological and biochemical analyses.

### Samples collection

Urine and stool samples were collected in 60 mL plastic screw-cap vials, and blood was collected in 5 mL dry tubes between 10.00 a.m. and 2.00 p.m. The samples were transported to the laboratory of the Divisional Hospital of Tibati or the Health district of Tignère for processing. In the laboratory, we determined hematuria in urine samples by naked eyes. We concluded to visible hematuria when blood was visible in the urine, coloring it pink, red, or dark brown. After that, each urine sample was agitated to ensure adequate dispersal of eggs, and 10 mL of urine was filtered through a Nucleopore® filter. The filters were examined by microscopy for the presence of schistosome eggs. Stool samples were examined by a single thick smear technique using a 41.7 mg Kato-Katz template. The number of eggs for each schistosome species was counted. The infection intensity was calculated and expressed as eggs per gram of feces (epg) for *S. mansoni* or eggs per 10 ml of urine (eggs/10 ml) for *S. haematobium*. Serum obtained from blood samples after coating was kept at − 20 °C and transported to the laboratory in an icebox for the assay.

### Evaluation of the level of transaminases

Serum activities of alanine aminotransferase (ALT) and aspartate aminotransferase (AST) were evaluated by colorimetric methods using Commercial Fortress kits (Fortress Diagnostics, UK) according to the protocol given by the manufacturer. Absorbances were read against the blank at 505 nm. The ALT and AST activities were determined from their respective standard curves with a lower limit of detection of 0.00 UI/L for ALT and AST and an upper limit of detection of 97 UI/L for ALT and 89 UI/L for AST.

### Testosterone assay

Concentrations of serum testosterone were evaluated by an enzyme immunoassay using the Human Testosterone ELISA kit (Invitrogen). A volume of 50 µL of standards and samples were dispensed in microtiter plates. After adding 100 µL of testosterone—HRP conjugate (testosterone labeled with horseradish peroxidase), plates were incubated for 60 min at room temperature. The competitive reaction was stopped by washing with wash buffer. Next, a chromogenic solution of 3-3′, 5-5′ tetramethylbenzidine (100 µL) was added to each well, and after incubation for 30 min at room temperature, 100 µL of the stop reagent (HCl 2 N) was added. Absorbance was read at 450 nm, and testosterone concentrations were determined from the standard curve with lower and upper limits of detection of 0.00 and 37.40 ng/mL, respectively.

### Data analysis

The data for this study were entered into Excel and exported to R version 3.5.0 software for statistical analysis. In univariate analysis, central tendency and dispersion measures were reported for quantitative variables, while the frequencies and percentages were written for the modalities of qualitative variables. In bivariate analysis, frequencies and percentages were determined for *Schistosoma*-positive (SCH-positive) and *Schistosoma*-negative (SCH-negative) subjects, respectively, to evaluate its prevalence across groups of other qualitative variables. Equality of prevalence rates was tested using Pearson’s chi-squared test for equality of proportions. Differences in distributions for quantitative variables between SCH-positive and SCH-negative subjects were tested using the Kolmogorov–Smirnov test. Univariate logistic regression models were used to identify factors affected by *Schistosoma* infection. For the quantitative results of biochemical parameters, data were analysed using GraphPrism 8.0.1 by one-way analysis of variance (ANOVA), and differences between groups were assessed using the Tukey multiple comparison post-test. In all, the level of significance was set at 5%. The intensity of individual infection for *S. mansoni* (light: 1–99 epg; moderate: 100–399 epg; and heavy ≥ 400 epg) and *S. haematobium* (light: < 50 eggs/10 mL and heavy: ≥ 50 eggs/10 mL) was classified according to the WHO guidelines [31].

## Results

### Sociodemographic characteristics of the participants

A total of 89 men of reproductive age (range 14–56 years) were enrolled in this study, with 51 in Tibati and 38 in Wouldé. The mean age of the population was 32.21 ± 1.16 years. Regarding the marital status of the participants, 53 (58.43%) were married, 32 (35.96%) were single with girlfriends, 4 (4.49%) were divorced, and 1 (1.12%) was widowed. The results revealed that 49.44% of the population was permanently in contact with schistosomiasis transmission sites (STS). Considering the study population per locality, 76.32% of the participants in Wouldé and 29.41% in Tibati were exposed to STS. (Table 1).

**Table 1:** Sociodemographic characteristics of the population of Tibati and Wouldé

Sociodemographic indicators	Category	Study areas	SCH-negative (n = 54)	SCH-positive (n = 35)	Total	Mean ± SEM/prevalence
Age (years)	Mean number		33.87	29.66	89	32.21 ± 1.16
Weight (kg)	Mean number		64.37	61.43	89	63.21 ± 0.89
Height (cm)	Mean number		169.22	166.17	80	167.89 ± 0.88
Profession	No frequent contact with STS	Tibati	21	15	36	70.59%
		Wouldé	2	7	9	23.68%
		Total	23	22	45	51.68%
	Frequent contact with STS	Tibati	12	3	15	29.41%
		Wouldé	19	10	29	76.32%
		Total	31	13	44	49.44%
Marital status	Single		16	16	32	35.96%
	Divorced		4	0	4	4.49%
	Married		34	18	52	58.43%
	Widowed		0	1	1	1.12%

Considering the indicator “profession,” the prevalence is the percentage of people by locality whose occupation exposes them or not to frequent contact with water

SCH-negative: negative to *Schistosoma* infection

SCH-positive: positive to *Schistosoma* infection

STS: schistosomiasis transmission sites

### Prevalence and intensity of infection

**Fig. 2:**
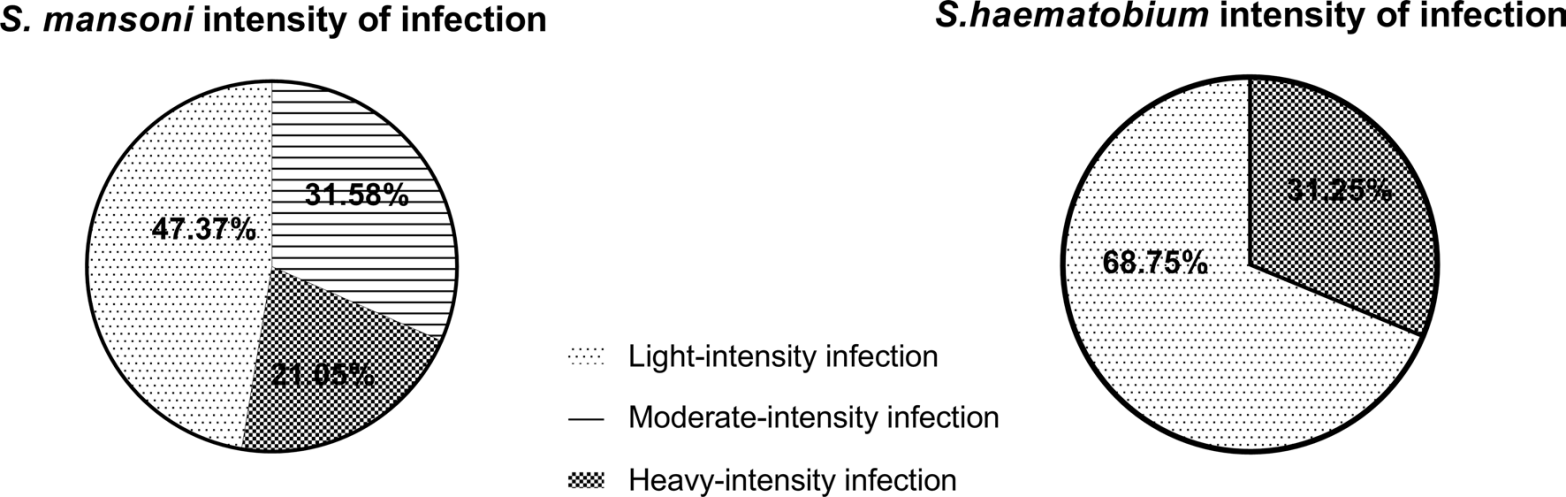
Degree of *Schistosoma* infection intensity in the adult male population of Tibati and Wouldé. Light-infection intensity: 1–99 epg; moderate-infection intensity: 100–399 epg; and heavy-infection intensity ≥ 400 epg for *S. mansoni* and for *S. haematobium*, light-infection intensity: < 50 eggs/10 mL and heavy-infection intensity: ≥ 50 eggs/10 mL

Table 2 summarizes the prevalence and intensity of *S. haematobium* and *S. mansoni* infection in the male of reproductive age involved in this study. *S. haematobium* was the most prevalent species in Tibati, with 31.37%, while the prevalence of *S. mansoni* was 3.92%. In Wouldé, *S. haematobium* was absent among the study population, and the prevalence of *S. mansoni* was 44.74%. The egg count for urogenital schistosomiasis ranged from 2 to 2620 eggs/10 mL and from 24 to 1224 epg for intestinal schistosomiasis. The geometric mean infection intensity was 22.12 ± 9.57 eggs/10 mL for *S. haematobium* and 128.10 ± 3.76 epg for *S. mansoni*. According to the degree of intensity of infection, 47.37% of *S. mansoni*-positive men presented light-intensity infection, 31.58% moderate-intensity infection, and 21.05% heavy-intensity infection. Light-intensity infection with *S. haematobium* represented 68.75%, while heavy-intensity infection was 31.25%. Visible hematuria was present in 80% of the men presenting *S. haematobium* heavy-intensity infection (Fig. 2).

**Table 2:** Prevalence and intensity of *Schistosoma* infection in the male adult population of Tibati and Wouldé

Localities	*Schistosoma haematobium*				*Schistosoma mansoni*			
	Number of subjects	Number of positive	Prevalence (%)	Intensity of infection (eggs/10 mL)	Number of subjects	Number of positive	Prevalence (%)	Intensity of infection (eggs/g)
Tibati	51 (57.30%)	16	31.37	22.12 (6.64 – 73.72)	51	2	3.92	24
Wouldé	38 (42.70%)	0	0	/	38	17	44.74	128.10 (67.66 – 242.60)

The geometric mean with 95% CI is used to express the intensity of infection

### Serum activity of transaminases

The serum activity of transaminases was evaluated in the study population. The alanine aminotransferase (ALT) and aspartate aminotransferase (AST) activities of *S. haematobium* and *S. mansoni*-positive men were close to SCH-negative men. There was thus no significant difference in the serum activity of transaminases between SCH-positive men and SCH-negative men (Table 3).

**Table 3:** Transaminases activities of *Schistosoma haematobium* and *Schistosoma mansoni*-positive individuals of Tibati and Wouldé

Transaminases	SCH-negative	*S. haematobium*-positive	*S. mansoni*-positive
ALT (UI/L)	12.84 ± 1.26	12.19 ± 1.66	13.80 ± 1.52
AST (UI/L)	42.03 ± 4.26	43.40 ± 4.70	40.27 ± 2.44

SCH-negative: negative to *Schistosoma* infection

ALT: alanine aminotransferase

AST: aspartate aminotransferase

### Testicular size and serum level of testosterone

**Fig. 3:**
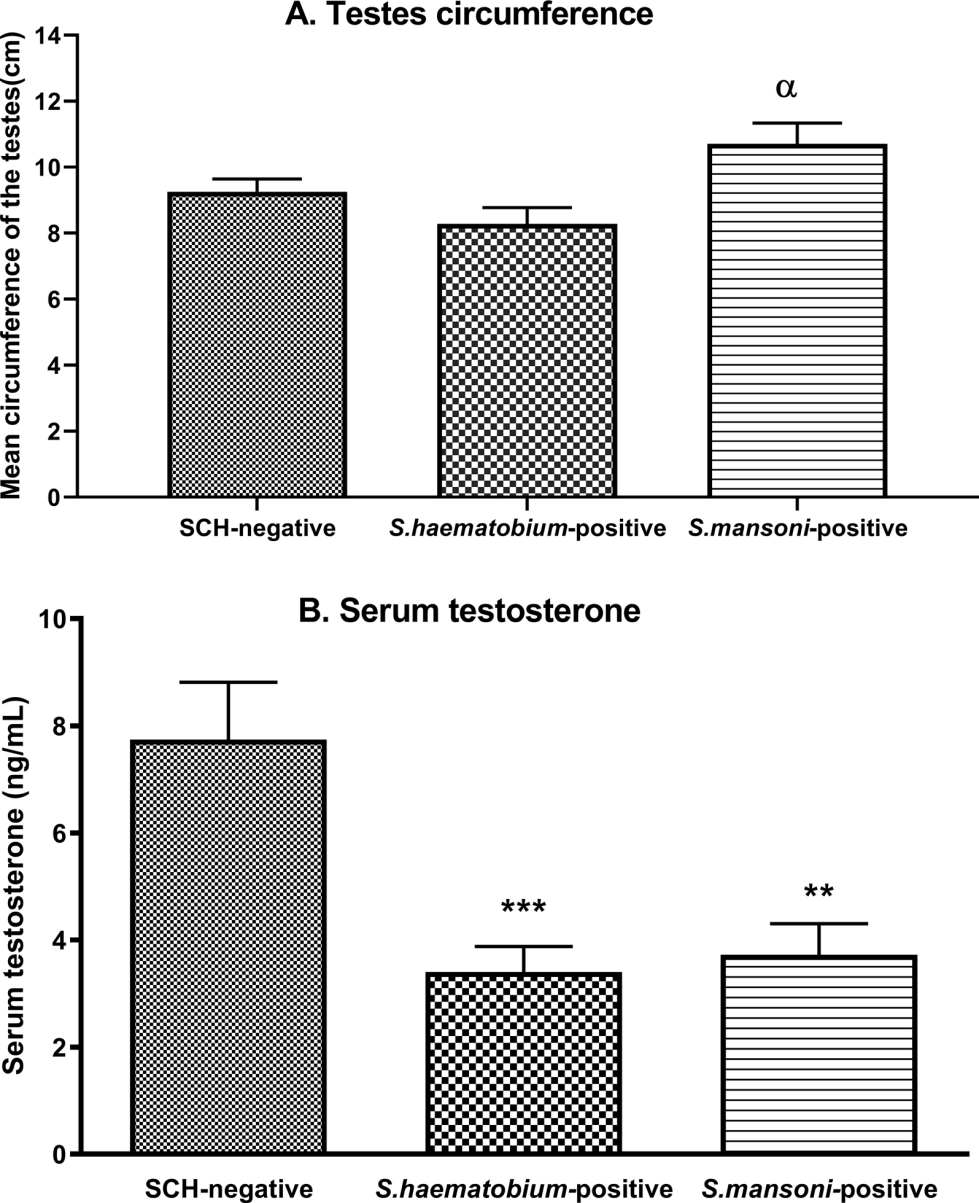
Mean testes circumferences (**A**) and serum testosterone levels (**B**) in *Schistosoma haematobium-* and *Schistosoma mansoni*-positive men in Tibati and Wouldé. Data are expressed as mean ± SEM. ANOVA followed by Tukey’s multiple comparison post hoc test was used for statistical analysis. ^**α**^*P* < 0.05: values are significantly different from those of *S. haematobium*-positive men. **P < 0.01, ***P < 0.001: values are significantly different from those of SCH-negative men

The mean testicular circumference of SCH-negative men was 9.26 ± 0.38 cm, and that of *S. haematobium-* or *S. mansoni*-positive men was 8.28 ± 0.49 cm and 10.71 ± 0.62 cm, respectively. These results revealed no significant difference between SCH-positive individuals and SCH-negative individuals. However, the testicular circumference of *S. mansoni*-positive men was higher than that of *S. haematobium*-positive men (*P* < 0.05) (Fig. 3a).

**Fig. 4:**
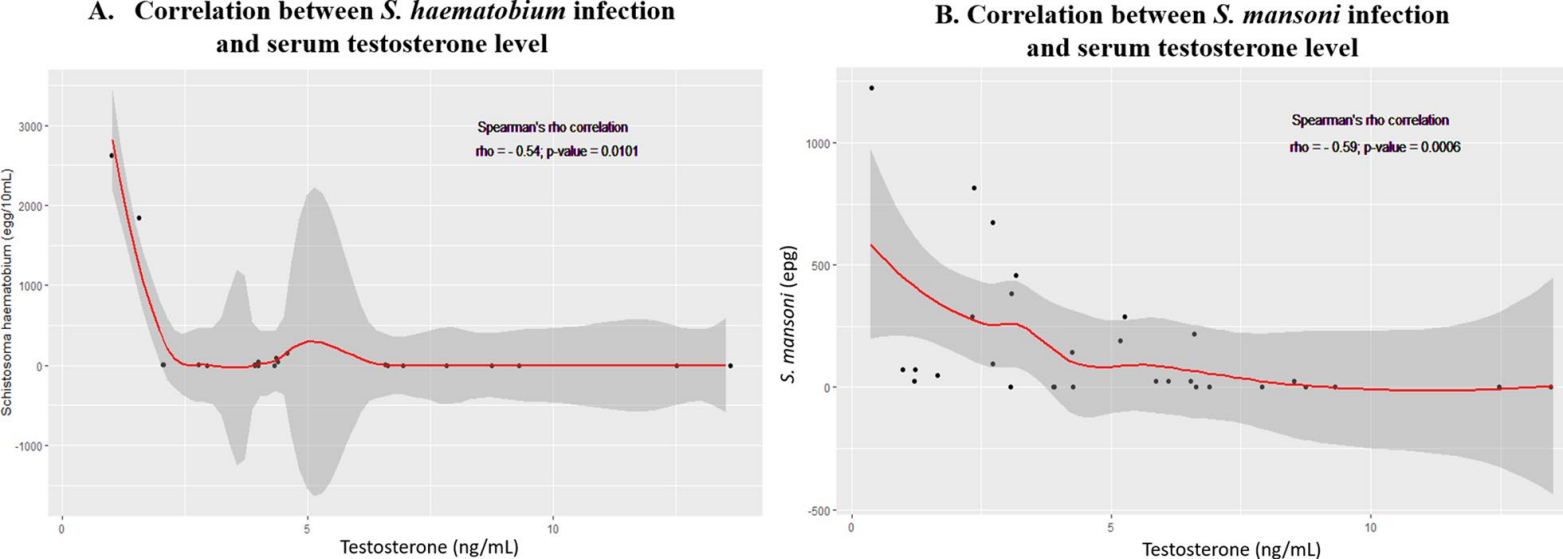
Correlation curves between *Schistosoma haematobium* (**A**) or *Schistosoma mansoni* (**B**) intensity of infection and serum testosterone levels

The serum concentrations of testosterone were 3.40 ± 0.48 ng/mL and 3.72 ± 0.58 ng/mL for *S. haematobium-* and *S. mansoni-*positive men, respectively, versus 7.74 ± 1.07 ng/mL for SCH-negative men. In comparison to the SCH-negative group, *Schistosom*a infection induced a significant reduction in the serum level of testosterone by 56.07% in the *S. haematobium*-positive group (*P* < 0.001) and by 51.94% in the *S. mansoni*-positive group (*P* < 0.01) (Fig. 3b). A strong correlation was observed between *Schistosoma* infection and the serum testosterone level in the study population. The Spearman’s rho correlation coefficients were − 0.54 (*P* = 0.0101) and − 0.56 (*P* = 0.0006) for the groups of *S. haematobium-* and *S. mansoni-*positive men, respectively (Fig. 4).

### Prevalence of genital tract pathologies

Table 4 shows the prevalence of genital tract pathologies in SCH-negative and SCH-positive men. Physical examination of the study population revealed that some men positive to *S. haematobium* or *S. mansoni* presented one or two of the following pathologies: varicocele, hydrocele, nodular epididymis, scrotal abnormalities, and inguinal hernia. Scrotal abnormalities were predominant with 25.00% and 52.63% of the cases in *S. haematobium*- and *S. mansoni*- positive men, respectively, versus 40.74% of patients in SCH-negative men. Varicocele and nodular epididymis were also detected in approximately 20.00% of SCH-positive men. These pathologies were also present in SCH-negative men. The bivariate logistic regression model results show that genital tract pathologies are unrelated to SCH infection: no significant difference was observed between SCH-negative and SCH-positive men.

**Table 4:** Prevalence of genital tract pathologies in *Schistosoma haematobium-* or *Schistosoma mansoni*-positive men of Tibati and Wouldé

Pathology	SCH-negative (n = 54)	*Schistosoma*-positive individuals							
		*S. haematobium* (n = 16)				*S. mansoni* (n = 19)			
	Proportion	Proportion	Odds ratio	CI (95%)	*P*-value	Proportion	Odds ratio	CI (95%)	*P*-value
Scrotal abnormalities	22 (40.74%)	4 (25.00%)	0.4848	0.1565–1.6790	0.3782	10 (52.63%)	1.6160	0.5820–4.6740	0.4267
Nodular epididymis	13 (24.07%)	2 (12.50%)	0.4734	0.0968–2.1130	0.4919	5 (26.32%)	1.3990	0.4457–4.568	0.7461
Varicocele	11 (20.37%)	3 (18.78)	0.9020	0.2407 –3.3764	> 0.9999	4 (21.05%)	1.0424	0.3250–3.9692	> 0.9999
Inguinal hernia	7 (12.96%)	1 (6.25%)	0.4387	0.0365–3.0033	0.6694	4 (21.05%)	1.8901	0.5449–7.9074	0.4508
Hydrocele	4 (7.41%)	1 (6.25%)	0.7678	0.05893–5.4633	> 0.9999	2 (10.53%)	1.6538	0.2865–7.8212	0.6257

SCH-negative: negative to *Schistosoma* infection

## Discussion

In schistosomiasis endemic areas, people are infected during their routine activities when exposed to contaminated water. In this study, approximately half of the population was exposed to schistosomiasis transmission sites due to their agricultural and fishing activities. Three-quarters of the study population in Wouldé was made up of farmers working in marshy fields around the Wouldé River. The study population in Wouldé was then a high-risk group for schistosomiasis infection [3]. The prevalence of *S. haematobium* or *S. mansoni* infection was within the interval of 10–49%, which reflects a moderate occurrence in the study population in Tibati or Wouldé. Despite this moderate endemicity of schistosomiasis, 80% of men with *S. haematobium* heavy-intensity infection presented visible hematuria. It reflects the increase in egg population in the urothelium and is sometimes linked to bladder and ureteral calcification and renal failure [32].

Among other complications of urogenital schistosomiasis, male reproductive function impairment and even infertility are quite common. *S. haematobium* is more involved than *S. mansoni* [33]. The symptoms include epididymitis, haemospermia, pain during urination, prostatitis, dilatation and calcification of seminal vesicles, and testicular inflammation [7, 34, 35]. In the current study, the mean testicular circumference of *S. mansoni*-positive men was significantly higher than that of *S. haematobium*-positive men. The increased size of the testes could presumably be the consequence of numerous eggs trapped in that organ or/and the inflammation induced by their presence in the testes. The presence of *S. haematobium* or *S. mansoni* ova in the testes has effectively been reported in men living in SCH-endemic areas or tourists who have visited these areas [14, 19, 21–23, 36]. *Schistosoma* ova trapped in the testes directly damages the testicular tissue through inflammation and granuloma formation [37], thus impairing testicular steroidogenesis. The testis is an exocrine and endocrine gland that synthesizes and produces testosterone through steroidogenesis in Leydig cells and sperm through spermatogenesis in the seminiferous tubules [38]. When *Schistosoma* eggs invade the testes, inflammatory cells migrate around eggs and generate granulomatous lesions. Lenzi et al. [39] described the bilharzial granuloma as a structure composed of macrophages, lymphocytes, eosinophils, neutrophils, giant cells, and fibroblasts surrounding schistosome eggs. The modulation of Leydig cell steroidogenesis by macrophages has been demonstrated [40–42]. Under normal physiological and noninflammatory conditions, testicular interstitial macrophages play an essential role in Leydig cell proliferation and differentiation and stimulate steroidogenic function [40, 41]. When macrophages are activated, they produce inflammatory cytokines such as tumor necrosis factor-alpha (TNF-α) and interleukin-1 (IL-1) that inhibit Leydig cell steroidogenesis and thus testosterone production. They also act as transcriptional repressors of steroidogenic enzyme gene expression [40, 43]. In fact, the exposure of TM3 Leydig cells to the inflammatory cytokines TNF-α, IL-1β, and IL-6 resulted in a decrease in Leydig cell viability and testosterone concentrations [42]. Moreover, the endocrine function of the testis is perturbed by the reactive oxygen species (ROS) generated by macrophages. They impair Leydig cell mitochondria by inhibiting steroidogenic acute regulatory (StAR) protein expression [40]. The significant decrease in testosterone concentration recorded during the current study in *S. haematobium*-positive men and *S. mansoni*-positive men could be linked to the inhibition of Leydig cell steroidogenesis by secretory products of granuloma inflammatory cells. In addition, a significant correlation between *S. haematobium* or *S. mansoni* infection and reduced testosterone levels was established in our study. Hormonal imbalance in SCH-infected individuals is less reported. Authors have recorded decreased levels of hydroxyprogesterone, testosterone, and dihydrotestosterone in boys and men presenting hepatosplenic schistosomiasis [26–29]. This was associated with an increased concentration of estradiol, estrone, and estriol [25, 29], suggesting an intense aromatization of androgens to estrogens in SCH-infected persons. Such sex steroid imbalance has delayed puberty in young boys and induced hypogonadism in men [27, 29].

Authors have described hydrocele, testicular atrophy, and nodular scrotum in patients presenting testicular schistosomiasis [21]. Therefore, this study denoted genital tract pathologies in SCH-positive men such as scrotal abnormalities, nodular epididymis, varicocele, hydrocele, and inguinal hernia. Since the prevalence of these pathologies was statistically comparable between SCH-positive and SCH-negative men, it would have become a challenge to establish a correlation between schistosomiasis or reduced testosterone levels and these pathologies. However, the testosterone level of SCH-negative men in this study was similar to that of a control group of men of reproductive age involved in a survey on the effect of agropesticides on the male reproductive function of farmers in a village in the West region of Cameroon [44].

The consequence of testosterone depletion in SCH-positive men in Tibati and Wouldé would be poor semen quality since spermatogenesis relies on the ability of Leydig cells to produce testosterone [38]. Unfortunately, due to traditional and cultural beliefs, it was impossible to conduct semen analysis in this study; the population of our study areas considered masturbation (method of sperm collection) as taboo. However, some authors have reported *Schistosoma* egg excretion in the semen, low volume of ejaculate, decreased sperm viability, oligoasthenoteratozoospermia, azoospermia, and sperm apoptosis [15–18, 20, 36, 45–47]. In addition, leukocytospermia, mainly eosinophilia, and secretion of inflammatory cytokines in the semen were also reported in SCH-positive men [15, 17, 20, 36, 45]. Therefore, a clinical scheme presenting a low testosterone level and/or poor semen quality could presumably be conducted for subfertility or infertility.

## Conclusion

This study demonstrated that *S. haematobium* and *S. mansoni* infections are associated with reducing the male reproductive hormone testosterone of men of reproductive age living in schistosomiasis endemic areas in Cameroon. The recommendations from this study are that the National Control Programme for Schistosomiasis and Intestinal Helminthiasis in Cameroon should accelerate the extension of schistosomiasis treatment to reach all individuals at risk in communities. In addition, a program of sensitization and surveillance of young people and adults suffering from male genital schistosomiasis should be set up in endemic areas.

## Data Availability

The datasets generated and analyzed during the current study are available from the corresponding author on reasonable request.

## Abbreviations

DALY: Disability-adjusted life years
FGS: Female genital schistosomiasis
MGS: Male genital schistosomiasis
ALT: Alanine aminotransferase
AST: Aspartate aminotransferase
ELISA: Enzyme-linked immunosorbent assay
HRP: Horseradish peroxidase
HCl: Hydrochloric acid
ANOVA: Analysis of variance
WHO: World Health Organization
SCH: Schistosomiasis
SCH-negative: Negative to *Schistosoma* infection
SCH-positive: Positive to *Schistosoma* infection
STS: Schistosomiasis transmission sites
TNF-α: Tumor necrosis factor-alpha
IL-1: Interleukin-1
IL-6: Interleukin-6
TM3 Leydig cells: Leydig cell lines derived from 11 to 13 days mouse testes
ROS: Reactive oxygen species
StAR: Steroidogenic acute regulatory
